# Cardiac autonomic responses induced by a single bout of exercise with flexible pole

**DOI:** 10.1186/1755-7682-7-40

**Published:** 2014-09-23

**Authors:** Ana Márcia dos Santos António, Marcelo Tavella Navega, Marco Aurélio Cardoso, Luiz Carlos Abreu, Vitor E Valenti

**Affiliations:** Centro de Estudos do Sistema Nervoso Autônomo (CESNA), Programa de Pós-Graduação em Fisioterapia, Faculdade de Ciências e Tecnologia, UNESP, Presidente Prudente, SP Brasil; Departamento de Fisioterapia e Terapia Ocupacional, Faculdade de Filosofia e Ciências, UNESP, Marília, SP Brasil; Departamento de Morfologia e Fisiologia, Faculdade de Medicina do ABC, Santo André, SP Brasil

**Keywords:** Cardiovascular system, Autonomic nervous system, Exercise therapy

## Abstract

**Background:**

Flexible poles are tools used to provide rapid eccentric and concentric muscle contractions. It lacks in the literature studies that analyze acute cardiovascular responses in different exercises performed with this instrument. It was investigated the acute effects of exercise with flexible poles on heart period in healthy women.

**Methods:**

The study was performed on 32 women between 18 and 25 years old. It was evaluated the heart rate variability (HRV) in the time (SDNN, RMSSD and pNN50) and frequency domain (HF, LF and LF/HF ratio). The subjects remained at rest for 10 minutes. After the rest period, the volunteers performed the exercises with the flexible poles. Immediately after the exercise protocol, the volunteers remained seated at rest for 60 minutes and HRV were analyzed.

**Results:**

It was observed no significance changes in the time domain (SDNN: p = 0.14; RMSSD: p = 0.8 and pNN50: p = 0.86) and frequency domain indices (LF (nu): 0.4; LF (ms^2^): p = 0.34; HF (nu): p = 0.4; HF (ms^2^): p = 0.8 and LF/HF ratio: p = 0.3) between before and after single bout of exercise with flexible pole.

**Conclusion:**

A single bout of exercise with flexible pole did not significantly change cardiac autonomic regulation in healthy women.

## Introduction

The effects of oscillatory motion on the human body have been studied for a long time. The stems are oscillatory intervention that allow quick eccentric and concentric muscle contractions, causing co-contraction of the muscle groups of the upper limb by means of oscillatory movements of the rod equipment [1]. The exercise with the oscillating rod is differentiated from other workouts vibration due to the lower frequency achieved ranging from 3 Hz to 4.5 Hz, causing effects on the musculoskeletal system [2, 3]. Some studies report that the rhythmic muscle contractions caused by oscillatory movements can alter some cardiovascular parameters [4–6].

Lister and his colleagues identified through surface electromyography, the supraspinatus muscles, trapezius fiber upper and lower trapezius fibers have greater activity during exercises performed with the use of vibrating rod than with elastic band or free charges [7]. Other studies have found a large activation of the stabilizing muscles of the trunk during exercise with the oscillating rod through electromyography [8, 9]. Evidence from electromyographic records show that activation of motor units of muscle fibers recruited during a contraction is related to the neural mechanism of central command, which requires immediate changes in the level of efferent activity of the sympathetic nervous system and parasympathetic nervous system acting on the heart, and the sympathetic nervous system acting on blood vessels [10, 11]. And the peripheral mechanism related to mechanical and metabolic activity of the muscle contraction initially transmitted by afferent fibers of muscle receptors of group III and IV and reach areas of cardiovascular control almost simultaneously to neural impulses from the central command [12, 13].

During exercise quick adjustments are needed in the cardiovascular system to maintain homeostasis, and such adjustments occur through the actions of the autonomic nervous system on the heart and blood vessels, depending on the type of exercise, intensity, duration and muscle mass involved [14].

Heart rate variability (HRV) is a noninvasive way the behavior of cardiac autonomic control and the heart’s ability to respond to various physiological and pathological stimuli [15]. This method evaluates the fluctuations in the intervals between consecutive heart beats (RR intervals) that are related to the influences of the autonomic nervous system on the sinus node. Furthermore, additional protocols for cardiovascular rehabilitation based on scientific evidence are always welcome.

The exercise with flexible pole is widely used in physical rehabilitation as it helps in the activation of the muscles of the upper limbs and trunk, and consequently in improving muscle strength and endurance, however, cardiac autonomic behavior in this type of exercise have not been studied. Thus, this study aims to examine the cardiac autonomic modulation after an exercise protocol with the flexible pole.

## Methods

### Study population

Subjects were 32 healthy female students, all nonsmokers, aged between 18 and 25 years. All volunteers were informed about the procedures and objectives of the study and gave written informed consent. All study procedures were approved by the Ethics Committee in Research of the Faculty of Sciences of the Universidade Estadual Paulista, Campus of Marilia (No. 0554–2012), and were in accordance with Resolution 196/96 National Health 10/10/1996.

#### Non-inclusion criteria

It was not included subjects that reported the following conditions: cardiopulmonary, psychological, neurological related disorders and other impairments that prevent the subject known to perform procedures, and treatment with drugs that influence cardiac autonomic regulation. Volunteers were not evaluated on 10–15 days and 20–25 days after the first day of the menstrual cycle. Where excluded physically active subjects according to the International Physical Activity Questionnaire (IPAQ) [16].

#### Initial evaluation

Prior to the study, baseline criteria included: age, gender, weight, height and body mass index (BMI). Weight was determined using a digital scale (W 200/5, Welmy, Brazil) with a precision of 0.1 kg. Height was determined using a stadiometer (ES 2020, Sanny, Brazil) with a precision of 0.1 cm and 2.20 m of extension. Body mass index (BMI) was calculated as weight/height^2^, with weight in kilograms and height in meters.

#### HRV analysis

The R-R intervals recorded by the portable RS800CX heart rate (HR) monitor (with a sampling rate of 1000 Hz) were downloaded to the Polar Precision Performance program (v. 3.0, Polar Electro, Finland). The software enabled the visualization of HR and the extraction of a cardiac period (R-R interval) file in “txt” format. Following digital filtering complemented with manual filtering for the elimination of premature ectopic beats and artifacts, at least 256 R–R intervals were used for the data analysis. Only series with more than 95% sinus rhythm was included in the study [17]. For calculation of the linear indices were used the HRV Analysis software (Kubios HRV v.1.1 for Windows, Biomedical Signal Analysis Group, Department of Applied Physics, University of Kuopio, Finland).

#### Linear indices of HRV

To analyze HRV in the frequency domain, the low frequency (LF =0.04–0.15 Hz) and high frequency (HF = 0.15–0.40 Hz) spectral components were used in ms^2^ and normalized units (nu), representing a value relative to each spectral component in relation to the total power minus the very low frequency (VLF) components, and the ratio between these components (LF/HF). The spectral analysis was calculated using the Fast Fourier Transform algorithm [18].

The analysis in the time domain was performed in terms of SDNN (standard deviation of normal-to-normal R-R intervals), pNN50 (percentage of adjacent RR intervals with a difference of duration greater than 50 ms) and RMSSD (root-mean square of differences between adjacent normal RR intervals in a time interval).

It was used Kubios HRV version 2.0 software to analyze these indices [19].

#### Protocol

Data collection was carried out in the same sound-proof room for all volunteers with the temperature between 21°C and 25°C and relative humidity between 50 and 60% and volunteers were instructed not to drink alcohol and caffeine for 24 hours before evaluation. Data were collected on an individual basis, between 8 and 12 AM to standardize the protocol. All procedures necessary for the data collection were explained on an individual basis and the subjects were instructed to remain at rest and avoid talking during the collection.

After the initial evaluation the heart monitor belt was then placed over the thorax, aligned with the distal third of the sternum and the Polar RS800CX heart rate receiver (Polar Electro®, Finland) was placed on the wrist. Before starting the exercises, the volunteers received visual feedback through a monitor to maintain neutral posture standing and were instructed to maintain the same posture throughout the exercise. Systolic and diastolic blood pressure was measured before, immediately after exercise and 60 minutes after exercise. The oscillatory movement of the flexible pole (Flexibar®) was held by flexion and elbow extension. The flexible pole vibrated at a frequency of 5 Hz, and the oscillation frequency of the flexible pole was based on an auditory stimulation through a metronome (Quartz Metronome®) calibrated at 300 bpm [20].

The exercises with the flexible pole were conducted with volunteers at standing position with feet apart (wide base) and shoulder flexion as the proposed position. To maintain the proper shoulder flexion in each upper limb it was used as a target visual feedback. All exercises were performed for 15 seconds with 50–60 seconds of rest between each exercise. Three repetitions were performed for each exercise [21]. The exercises were performed with both arms on three positions: 1) with shoulders at approximately 180° of flexion with the flexible pole on the frontal plane, parallel to the ground , 2) with the shoulder on 90° of flexion with the flexible pole on the transverse plane , and 3) shoulders at 90° of flexion with the flexible pole on the sagittal plane, perpendicular to the ground. HRV was analyzed at the following periods: control rest, 0–20 min, 20–40 min, and 40–60 min after the protocol exercise.

#### Statistical analysis

Standard statistical methods were used to calculate the means and standard deviations. The Shapiro-Wilk test was performed to evaluate the distributions. For parametric distributions we applied ANOVA for repeated measures followed by the Bonferroni posttest. For non-parametric distributions we used the Friedman test followed by Dunn’s posttest. Differences were considered significant when the probability of a Type I error was less than 5% (p < 0.05). We used Biostat 2009 Professional 5.8.4 software.

## Results

Data on baseline systolic (SAP) and diastolic arterial pressure (DAP), heart rate (HR) and mean RR interval, age, height, body weight and body mass index (BMI) are presented in Table 1.

**Table 1:** **Baseline heart rate (HR), mean RR interval (Mean RR), age, weight, height and body mass index (BMI) of the volunteers**

Variable	Value
Height (m)	1.63 ± 0.06
Age	19.7 ± 1.8
Weight (kg)	59.5 ± 9
BMI (kg/m^2^)	22.28 ± 3.1
HR (bpm)	80.11 ± 10
Mean RR (ms)	811.5 ± 97

m: meters; kg: kilograms; bpm: beats per minute; ms: milliseconds.

It was observed that the flexible pole exercise protocol did not induce changes in diastolic and systolic arterial pressure (Table 2).

**Table 2:** **Diastolic (DAP) and systolic arterial pressure (SAP), time and frequency domain indices before and after exercise with flexible pole**

Variable	Rest	0-20 min	20-40 min	40-60 min	p
**SAP (mmHg)**	109.0 ± 9	109.0 ± 8	-	-	0.9
**DAP (mmHg)**	68.6 ± 1	63.3 ± 5	-	-	0.8
**LF (ms** ^**2**^ **)**	748.6 ± 529	859 ± 522	865 ± 439	832 ± 425	0.34
**HF (ms** ^**2**^ **)**	205 ± 97	390 ± 140	543 ± 225	219 ± 137	0.8
**LF (nu)**	75.4 ± 7	70.2 ± 13	78.7 ± 10	71.1 ± 11	0.4
**HF (nu)**	33.1 ± 13	20.8 ± 10	21.1 ± 10	28.6 ± 11	0.4
**LF/HF**	3.4 ± 1	4.7 ± 2	4.6 ± 2	3.1 ± 2	0.3
**SDNN**	46.6 ± 14	51.3 ± 13	52.8 ± 11	55.9 ± 16	0.14
**RMSSD**	30.7 ± 15	28.4 ± 12	29.0 ± 12	31.3 ± 15	0.8
**pNN50**	11.1 ± 1	9.9 ± 1	10.3 ± 1	12.3 ± 1	0.86

LF: Low frequency; HF: High frequency; LF/HF: Low frequency/High frequency ratio; SDNN: standard deviation of normal-to-normal R-R intervals; pNN50: percentage of adjacent RR intervals with a difference of duration greater than 50 ms; RMSSD: root-mean square of differences between adjacent normal RR intervals in a time interval. Mean ± Standard Deviation. ms: milliseconds; mmHg: millimeters of mercury.

In relation to the indices in the time domain, it was observed that the SDNN index, representing global variability of heart rate, was not significantly changed after the exercise protocol. Moreover, the parasympathetic components of HRV, pNN50 and RMSSD, were not significantly altered in the recovery period after the standardized protocol. Regarding the frequency domain indices of HRV, no significant changes were found for LF and HF in absolute and normalized units as well as for the LF/HF ratio between before and after the exercise protocol with flexible pole (Table 2).

## Discussion

Flexible pole exercises lead to isometric contraction of the shoulder and trunk muscles and isometric handgrip and isometric leg extension were shown to induce cardiovascular responses featured by increase in skin sympathetic nerve activity [22].

However, it lacks in the literature studies that investigated the effects of exercise with this tool on cardiovascular system. In this sense, this study aimed to investigate the acute effects of a single bout of exercise with flexible pole on cardiac autonomic regulation in healthy adult women. To determine the total sample it was previously done a pilot study with 10 people, which revealed significant results. This study observed no change in systolic and diastolic arterial pressure and HRV indices in response to the exercise protocol used in this study. Firstly, it was hypothesized significant responses of arterial blood pressure induced by the flexible exercise protocol. The post-exercise hypotension is defined as a decrease of blood pressure compared to the pre-exercise baseline levels [23]. Another study observed that the arterial blood pressure was increased in young men during static exercise at 40% of maximal voluntary contraction [24, 25].

The literature supports this result indicating that activation of the muscle chemoreflex during sustained isometric contractions increases blood pressure through increase in muscle sympathetic nerve activity [26]. However, based on the present results, there was no change in systolic and arterial pressure recovery after one bout of flexible pole exercise. It is believed that the intensity of the protocol exercise used in this study, that was based on previous studies, rather than the style of exercise, was the main reason for the absence of significant cardiovascular responses during the recovery phase [27].

As a main finding in this study, this study reported that a single bout of exercise with flexible pole did not promote significant cardiac autonomic responses, observed absence of parasympathetic changes induced by a single bout of the proposed exercise through analysis of the RMSSD and pNN50 time domain indices and HF in absolute and normalized unit index in the frequency domain. Engaging in physical effort induces an acutely increased risk of sudden cardiac death immediately following the period of activity and also during the period of exercise performance. The maintenance of HRV after the intervention proposed in this study supports the safety of this protocol of flexible pole exercise for patients with cardiovascular disorders. However, additional studies on this cardiovascular disease population are indicated before prescription.

The hypothesis of this study was to find significant responses of cardiac autonomic regulation induced by a protocol of exercise with flexible pole. Nonetheless, was found no significant changes in HRV indices recovery in the first hour after a single session of exercise. The central command and muscle chemoreflex are two mechanisms proposed to explain the usual pressor response to exercise. The muscle chemoreflex is a reaction mechanism activated by chemosensitive afferent nerve fibres situated in the exercising muscles. The central command is an efferent response triggered by parallel activation of the cardiovascular control centres and the motor cortex [28]. Immediately after exercise end, there is an interruption of inputs from the central nervous system and from the receptors in skeletal muscle, promoting an abrupt exponential fall of the heart rate, due to the vagal reactivation. On the other hand, this classical response was not observed in this study possibly due to the intensity of exercise that was based on auditory stimulation through a metronome calibrated at 300 bpm.

The intensity of flexible pole exercise proposed in this study was based on previous studies that used the oscillation frequency of the flexible pole on an auditory stimulation through a metronome calibrated at 300 bpm. A proposed way to control exercise intensity with flexible pole is modulating the rhythm of the auditory stimulation, i.e. higher intensities for higher bpm. Wilson et al. investigated cardiovascular and autonomic variables during isometric handgrip and isometric leg extension exercises [29]. The authors observed that the sympathetic increase induced by the exercises is dependent on the exercise intensity and that the magnitude of the exercise was not suggested to be associated with the amount of muscle mass involved or the exercising limb. In this context, it is possible that increase in the intensity of exercise with flexible pole by increasing the frequency of the auditory stimulation could increase cardiac autonomic recovery responses.

There is not a consensus in the literature confirming flexible pole protocol as a moderate exercise because it depends on the procedure used. In this study all exercises were performed for 15 seconds with 50–60 seconds of rest between each exercise and three repetitions were performed for each exercise. The exercises were performed with both arms on three positions: 1) with shoulders at approximately 180° of flexion with the flexible pole on the frontal plane, parallel to the ground, 2) with the shoulder on 90° of flexion with the flexible pole on the transverse plane 3) shoulders at 90° of flexion with the flexible pole on the sagittal plane, perpendicular to the ground. It may be hypothesized that if exercise performance were changed to 30 seconds instead of 15 seconds cardiac autonomic responses would be more intense.

The literature indicated differences between men and women regarding cardiac autonomic recovery after a section of exercise [30]. In order to avoid sex-dependent effects on cardiac autonomic responses induced by exercise it was investigated only women. In addition, the menstrual cycle was also showed to influence baseline nonlinear properties of HRV. In order to exclude the influence of the follicular and luteal phases of the menstrual cycle on cardiac autonomic regulation it was not evaluated volunteers on 10–15 days and 20–25 days after the first day of the menstrual cycle [31].

Flexible pole has been extensively used as an auxiliary tool to improve shoulder muscles physical capacities in physical therapy rehabilitation programs. On the other hand, after a careful review on Medline/Pubmed database, it was revealed that this is the first study to investigate the effects of a single bout of flexible pole exercise on cardiac autonomic modulation in healthy adult women. Considering that intense changes in cardiac autonomic regulation induced by exercises can lead to cardiac events such as sudden death [32], it is plausible to investigate the possibility of suggesting this type of exercise to patients with cardiovascular disorders. However, we suggest further acute and follow-up investigations to verify the training effect with flexible pole exercises in the cardiac overload and cardiopulmonary capacity of those patients.

## Conclusion

A single exercise session with a flexible pole did not induce cardiac autonomic responses in healthy adult women. It is believed that due to its low-intensity exercise, it was not able to cause a significant change in HRV. It is suggested further studies where the same protocol should be submitted in other groups of different ages to see if get the same answer obtained in this study.
